# Mycotic Aneurysm of the Superior Mesenteric Artery due to Infective Endocarditis

**DOI:** 10.1590/0037-8682-0049-2025

**Published:** 2025-05-09

**Authors:** Elif Gündoğdu, Mustafa Kürşat Topal

**Affiliations:** 1 Eskişehir Osmangazi University, Faculty of Medicine, Department of Radiology, Eskişehir, Turkey.

A 45-year-old male patient presented to the emergency department with a 1-month history of cough, fever, chills, sweating, and, recently, general malaise and fatigue. The patient's medical history included hypertrophic cardiomyopathy. The patient’s vital signs were within normal limits. Laboratory tests revealed leukocytosis (30.71×10³ uL) and elevated levels of C-reactive protein (99.1 mg/L), procalcitonin (12.60 ng/mL), and Troponin T (0.055 ng/mL). Echocardiography revealed a 42×18 mm vegetation originating from the mitral valve. Blood cultures revealed *Streptococcus oralis*. The patient was diagnosed with infective endocarditis and underwent mitral valve replacement. On the 15th postoperative day, the patient complained of periumbilical pain and abdominal computed tomography (CT) was performed. The CT scan showed an aneurysm approximately 3 cm in diameter distal to the superior mesenteric artery, with surrounding inflammatory soft tissue densities and fat tissue stranding (Figure 1). Mycotic aneurysms (MAs) are balloon-like dilatations of the arterial wall due to infection, usually bacterial^1^. MAs are associated with high mortality rates due to their increased risk of rupture and embolus production. CT is the modality of choice for detection. MAs appear on CT as lobulated vascular masses with an indistinct, irregular arterial wall and perivascular soft tissue inflammation and edema^2^. The noninvasive and rapid nature of CT allows for early diagnosis and intervention, significantly increasing the patient's chances of survival.

**FIGURE 1:**
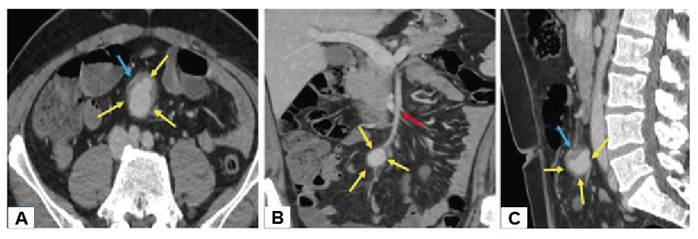
Axial **(A)**, coronal **(B)**, and sagittal **(C)** plane abdomen CT shows aneurysm (yellow arrows) of the superior mesenteric artery (red yellow) and perivascular fat stranding and inflammatory soft tissue (blue arrows).

## References

[1] Pérez Baztarrica G, Cherjovsky R, Blanco N, Porcile R (2009). Mycotic axillary artery aneurysm. Rev Esp Cardiol.

[2] Zhang N, Xiong W, Li Y, Mao Q, Xu S, Zhu J, Sun Z, Sun L (2021). Imaging features of mycotic aortic aneurysms. Quant Imaging Med Surg.

